# Contraceptive use and counselling in women with mental illness: KwaZulu-Natal, South Africa

**DOI:** 10.4102/sajpsychiatry.v31i0.2397

**Published:** 2025-06-04

**Authors:** Lisa Peralta, Reyanta Bridgmohun, Thandokazi Mcizana

**Affiliations:** 1 Department of Psychiatry, Faculty of Health Sciences, University of KwaZulu-Natal, Durban, South Africa; 2 Division of Actuarial Science, School of Management Studies, University of Cape Town, Cape Town, South Africa

**Keywords:** contraception, contraceptive counselling, mental illness, female, psychiatry, South Africa

## Abstract

**Background:**

While women with mental illness are prone to unplanned pregnancies, sexual violence and exposure to teratogenic medications, there is limited knowledge of contraceptive use and counselling in this vulnerable group.

**Aim:**

This study aims to determine the prevalence of contraceptive use and counselling in women of childbearing age attending a psychiatric facility in KwaZulu-Natal Province, South Africa.

**Setting:**

This study was conducted on in- and outpatients attending Townhill Hospital, a tertiary psychiatric facility in Pietermaritzburg that provides specialised services for the uMgungundlovu District and surrounds.

**Methods:**

An interviewer-designed and administered questionnaire was used to obtain data from 186 participants in this quantitative, cross-sectional study. Clinical information was obtained from the participants’ medical records.

**Results:**

Among the 186 participants, the prevalence of consistent contraceptive use was 50%, 65.9% of prior pregnancies were unplanned, 35.5% reported a history of forced sex, 25.8% reported having unmet contraceptive needs and 59.7% requested integrated health care services. Only a quarter (25.3%) reported having received contraceptive counselling from their mental health care practitioner, while 31.2% (*n* = 58) received counselling on medication teratogenicity. Both contraceptive counselling (*p* = 0.018) and teratogenicity counselling (*p* = 0.007) were significantly associated with contraceptive use (*n* = 111).

**Conclusion:**

There is inconsistent contraceptive use and low levels of counselling among women with mental illness. Integrated health care and contraceptive counselling by mental health care practitioners could improve the consistency of contraceptive use in this vulnerable group.

**Contribution:**

Contraceptive counselling should be incorporated into psychiatric services to increase contraceptive uptake and reduce the impact of unplanned pregnancy and teratogenicity in this vulnerable population.

## Introduction

Mental illness (MI) affects an estimated 970 million people worldwide, with women comprising the majority.^1,2^ Women of childbearing age with MI are a vulnerable population who require active contraceptive counselling and prescription in order to prevent unplanned pregnancies.^3,4^ Women with MI are often victims of sexual violence and are at increased risk for contracting sexually transmitted infections (STIs), specifically human immunodeficiency virus (HIV).^5,6^ Exposure of reproductive-age women to teratogenic psychiatric medications has been linked to poorer obstetric and neonatal outcomes,^7^ making contraceptive use essential in this at-risk population.

The United Nations State of the World Population 2022 report revealed that almost half of all pregnancies worldwide are unintended, a quarter being in Africa.^8^ Women with MI have an even higher frequency of unintended pregnancies and lower utilisation of consistent contraception compared to the general population.^8,9^ Psychiatric diagnosis-related factors, including impaired decision-making, impulsivity and vulnerability to sexual violence, further increase the risk for unplanned pregnancy and STIs.^10^ The impact of unplanned pregnancies is considerable and has been directly linked to poorer maternal mental health and increased maternal deaths.^8,11,12,13^

Unplanned pregnancies and their associated consequences can be prevented through consistent contraception use. Globally, approximately 56% of reproductive-age women use contraception, the rates in high-income countries being double those in their low-income counterparts.^14^ The South African Demographic and Health Survey 2016 found that the prevalence of contraceptive use in women aged 15–49 years was in keeping with global percentages at 58%.^15^ Injectable contraceptives are most frequently used, while long-acting methods are used less than 5% of the time.^15^ Globally, long-acting and permanent contraceptive methods, such as implants, intra-uterine contraceptive devices (IUCDs) and sterilisation, are preferred, while less permanent methods with a higher failure rate are often selected in sub-Saharan Africa.^15,16^

There are numerous barriers to contraceptive use.^17^ The social barriers include poor contraceptive knowledge, stigma and the patient’s religious and cultural views,^18^ while higher levels of education have been associated with an increased prevalence of use.^15,17^ Contraceptive side effects and the misperception of these have been implicated in the low uptake of their use,^19^ while peer and partner opinions have also been identified as a barrier, as women fear intimate partner violence when using them.^20^

Hormonal contraceptives are controversial in the context of MI because of their potential implication in mood disorders although the literature largely disputes this.^21,22^ Randomised, placebo-controlled studies have found that mood symptoms are less frequently or similarly reported in patients using hormonal contraceptives compared to non-users.^13^ However, research has revealed that mental health side effects, including mood swings and irritability, account for a large proportion of discontinuation in this population.^23^ Drug–drug interactions between psychotropic medications and hormonal contraceptives, including oral contraceptives increasing clozapine levels and carbamazepine reducing contraceptive efficacy, may result in potential adverse effects and contraceptive failure.^13,24^

Women with MI are also susceptible to low contraceptive uptake because of factors related to their MI, including impaired insight and cognitive deficits, affecting their decision-making and awareness of the need for contraception.^25,26^ Impulsivity, hypersexuality and comorbid substance use contribute to risky sexual behaviours, resulting in unplanned pregnancies, STIs and poor contraceptive adherence.^13,24^

Consistent contraceptive use is further necessitated in women with MI because of exposure to potentially teratogenic psychotropic medications. The teratogenicity of mood stabilisers, especially sodium valproate, carbamazepine and lithium, is well established.^27,28,29,30^ The supposedly safer alternatives, such as lamotrigine, have also been associated with foetal developmental anomalies.^30^ Exposure to other commonly prescribed psychiatric agents, including antidepressants and antipsychotics, is also not without risk, although they are generally considered safe because of a lack of conclusive evidence of teratogenicity.^27,31^

It is encouraged in psychiatric practice that women with MI should be adequately informed of their contraceptive options, the need to plan families and the potential teratogenicity of their medications.^32^ Despite this, very few South African or international studies exist on the current practices and impact of contraceptive counselling in patients with MI. An Ethiopian qualitative study found that although women with serious MI are at risk for sexual exploitation and unplanned pregnancies, they had not received contraceptive counselling from mental health care providers (MHCPs) and preferred integrating family planning into their mental health care services.^25^

In a cross-sectional study conducted in Soweto, South Africa, in which contraceptive use and family planning counselling in women with MI were explored, the prevalence of consistent contraceptive use was only 44.7%, with barrier contraceptive methods being used most frequently.^26^ Single participants used contraception less than those in a relationship, while those with depressive disorders used them more consistently. Family planning education was reported in only 26.8% of participants and was not significantly associated with contraception use.^26^

Aside from this single-centre study, no additional research on contraceptive use and counselling among women with MI in South Africa was found. Without sufficient information on the scope of contraceptive use, counselling and barriers to their use in women with MI, interventions cannot be appropriately directed to address the care gaps. The aim of this study was therefore to determine the prevalence of contraception use and counselling among women of childbearing age with MI in KwaZulu-Natal (KZN) Province, South Africa.

## Objectives

The primary objective was to determine the prevalence of contraception use and counselling in reproductive-age women with MI and any associations between these factors. The secondary objectives included determining the prevalence of unplanned pregnancy and teratogenic medication prescription.

## Research methods and design

### Study design, setting and participants

This quantitative, cross-sectional study was conducted at a tertiary-level, government psychiatric hospital in KZN, where patients are referred for specialised care from across the province. Data were collected from 01 August to 03 November 2023 using a convenience sampling method to identify 186 women from the outpatient department and inpatient female wards. The inclusion criteria were women aged 18–49 years who were psychiatrically stable and able to willingly consent. A minimum age of 18 years was determined because of the minimum age for consent and follow-up in the adult outpatient service. The limit of 49 years was regarded as the upper reproductive age in the literature, with post-menopausal women within the age range being excluded.

**FIGURE 1:**
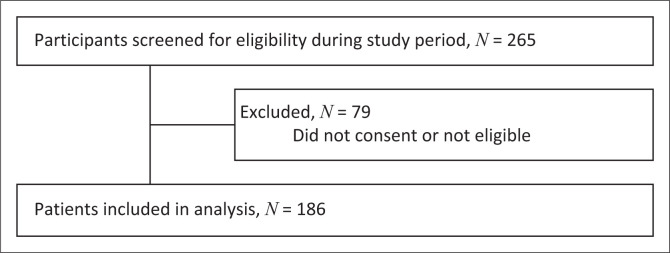
Participant selection process.

Participants were screened and enrolled in the study after obtaining their informed consent for the survey and chart review. A total of 265 women were screened for eligibility, and after excluding those who did not consent and were not eligible, 186 women were enrolled, comprising 126 outpatients and 60 inpatients (Figure 1).

### Data collection

Owing to a lack of standardised and validated tools for this purpose, a questionnaire was researcher-designed and piloted on five patients, with changes made thereafter to improve its use. Relevant demographic, reproductive, contraception and health care-experience information was collected by the principal investigator, and, where necessary, a trained isiZulu-speaking, professional psychiatric nurse was used as an interpreter when the participants chose to speak in their mother tongue. Thereafter, a brief retrospective chart review was conducted to obtain information pertaining to diagnoses, pharmacological management and evidence of contraceptive counselling and/or prescription.

### Definitions

Contraception refers to the prevention of pregnancy through the use of devices, medications, procedures or behaviours, with contraception and family planning being used interchangeably, and the methods available are categorised in Table 1.

**TABLE 1:** Contraceptive methods.

Duration of action	Hormonal	Non-hormonal
Short-acting	Oral contraceptive pill	Male condom
	Patch	Female condom, diaphragm
	Two- or three-month injection	Behavioural methods (i.e. fertility awareness, withdrawal)
	Emergency – Morning-after pill	Spermicides
Long-acting	Intra-uterine contraceptive device (i.e. Mirena)	Intra-uterine contraceptive device – Copper-T device
	Implanon	Tubal ligation
		Partner vasectomy

*Source:* Adapted from World Health Organization. Family planning/contraceptive methods [homepage on the Internet]. 2023 [cited 2024 Jun 09]. Available from: https://www.who.int/news-room/fact-sheets/detail/family-planning-contraception

The overall prevalence of contraceptive use was defined as the percentage of participants who reported current use of contraception at the time of the study. The prevalence of *consistent* contraception was defined as the percentage of participants who reported that they *always* use contraception (i.e. with each sexual encounter). The terms ‘unplanned’ and ‘unintended’ pregnancy were used interchangeably. Teratogens were defined as medications that cause an abnormality (physical or functional) to the developing foetus following exposure during pregnancy.^33^ The teratogenic medications included in the study were mood stabilisers (lithium, carbamazepine, sodium valproate and topiramate), methylphenidate and benzodiazepines.

### Data analysis

Data were captured on hard-copy forms and entered into an electronic database system, where it was stored and numerically coded for anonymity. Statistical analysis was undertaken using R Statistical Software using descriptive statistical methods. A chi-square (χ^2^) test was used to compare differences between groups and categorical variables, the Fisher’s exact test was used for categorical variables in 2 × 2 tables and the Wilcoxon Rank-Sum test was used for continuous numerical variables; the level of significance was set at *p* = 0.05.

### Ethical considerations

Ethical approval was obtained from the University of KwaZulu-Natal Biomedical Research Ethics Committee (BREC/00005114/2022) and Townhill Hospital Research Board, and the findings were circulated to the facility on completion. Informed consent was obtained from all participants, and their medical files were made accessible. No identifying data were captured to ensure anonymity, and a distress protocol and referral system for participants requesting contraception were put in place for them to access at their earliest convenience.

## Results

### Socio-demographic characteristics

Of the 186 participants, the majority were sourced from the outpatient department (67.7%, *n* = 126) (Table 2), the median age was 33 years (IQR 26–40), and most fell within the 30–39 years age category (38.2%, *n* = 71). Over half of the participants were Black Africans (53.2%, *n* = 99), with English (47.8%, *n* = 89) and isiZulu (41.4%, *n* = 77) being the most frequently spoken first languages. Over two-thirds were unemployed (70.4%, *n* = 131), while the majority had a grade 12 or higher level of education (74.7%, *n* = 139), and most were single (66.7%, *n* = 124).

**TABLE 2:** Comparison of socio-demographic and reproductive characteristics of inconsistent and consistent contraceptive users.

Variable	Overall (*N* = 186)		Inconsistent users (*N* = 93)		Consistent users (*N* = 93)		*P*
	*n*	%	*n*	%	*n*	%	
**Age (years)**	-	-	-	-	-	-	0.072
18–29	67	36.0	34	36.6	33	35.5	-
30–39	71	38.2	29	31.2	42	45.2	-
40–49	48	25.8	30	32.3	18	19.4	-
**Location**	-	-	-	-	-	-	0.018*
Inpatient	60	32.3	38	40.9	22	23.7	-
Outpatient	126	67.7	55	59.1	71	76.3	-
**Race**	-	-	-	-	-	-	0.092
Black	99	53.2	58	62.4	41	44.1	-
White	51	27.4	21	22.6	30	32.3	-
Indian	19	10.2	8	8.6	11	11.8	-
Mixed	17	9.1	6	6.5	11	11.8	-
**Language**	-	-	-	-	-	-	0.044*
English	89	47.8	37	39.8	52	55.9	-
isiZulu	77	41.4	46	49.5	31	33.3	-
isiXhosa	6	3.2	5	5.4	1	1.1	-
Afrikaans	11	5.9	4	4.3	7	7.5	-
Other	3	0.0	1	0.0	2	0.0	-
**Employed**	55	29.6	25	26.9	30	32.3	0.521
**Education**	-	-	-	-	-	-	0.805
No Matric	47	25.3	22	23.7	25	26.9	-
Matric	72	38.7	38	40.9	34	36.6	-
Tertiary	67	36.0	33	35.5	34	36.6	-
**Marital status**	-	-	-	-	-	-	0.049*
Single	124	66.7	66	71.0	58	62.4	-
Married	38	20.4	18	19.4	20	21.5	-
Cohabiting	7	3.8	1	1.1	6	6.5	-
Divorced	12	6.5	4	4.3	8	8.6	-
Widowed	4	2.2	4	4.3	-	0.0	-
Other	4	2.2	4	4.3	-	0.0	-
**Religion**	-	-	-	-	-	-	0.592
Christian	148	79.6	74	79.6	74	79.6	-
Hindu or Islam	14	7.5	6	6.5	8	8.6	-
Traditional	5	2.7	4	4.3	1	1.1	-
None or Other	19	10.2	9	9.7	10	10.8	-
**Sexually active**	103	55.4	40	43.0	63	67.7	0.001*
**Prior pregnancy**	123	66.1	54	58.1	69	74.2	0.029*
**Forced sex**	66	35.5	26	28.0	40	43.0	0.046*
**Desires children**	85	45.7	49	52.7	36	38.7	0.077

* , *p*-value statistically significant (*p* ≤ 0.05).

### Reproductive characteristics

Of the 186 participants, 55.4% reported being sexually active in the 6 months prior to the survey (*n* = 103). Two-thirds (66.1%, *n* = 123) reported having had a prior pregnancy, of which 65.9% were unplanned (*n* = 81), while 15.4% with prior pregnancies had a termination of pregnancy (*n* = 19). The prevalence of forced sex was 35.5% (*n* = 66), and less than half wanted future pregnancies (45.7%, *n* = 85).

### Contraception

The prevalence of current contraceptive use was 59.1% (*n* = 110), and the prevalence of consistent contraception use was 50% (*n* = 93). In the ‘inconsistent’ use group, 34% reported either ‘sometimes’ (*n* = 63) or ‘never’ (16%, *n* = 30) using contraception.

## Methods

**FIGURE 2:**
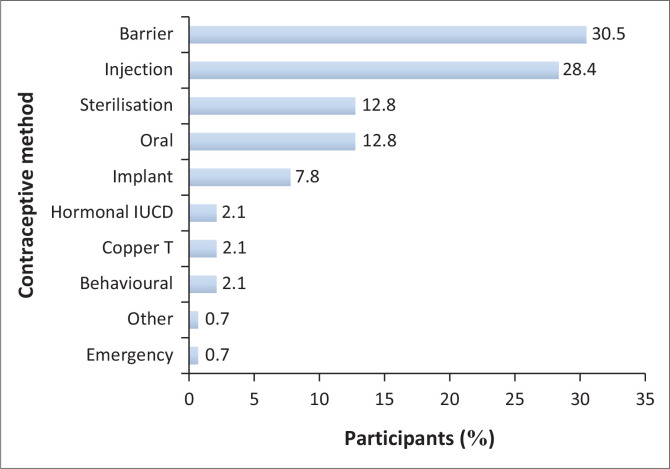
Contraceptive methods used by participants (%).

Barrier contraceptive methods were most frequently used (30.5% *n* = 42), with 28.4% using male condoms (*n* = 40) (Figure 2) and 28.4% relying on two- or three-monthly injectable contraceptives (*n* = 40); 12.1% reported combining male condoms with another method (*n* = 17), while 59% (*n* = 72) used hormonal contraceptives, and 71.3% reported using short-acting methods (*n* = 87). A minority (12.7%) relied on sterilisation, including tubal ligations or a partner’s vasectomy (*n* = 18).

### Barriers to contraception use

**FIGURE 3:**
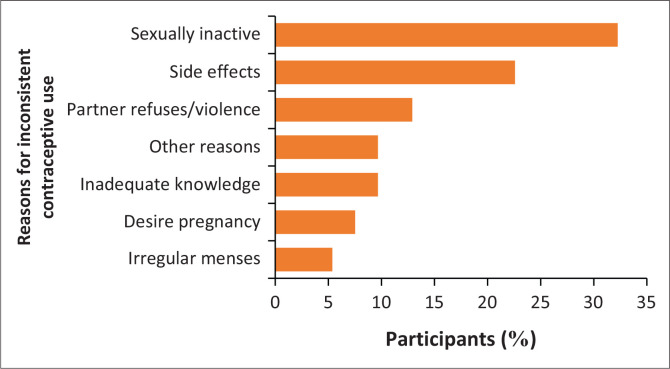
Reasons for inconsistent contraceptive use (%).

Sexual inactivity accounted for the majority of inconsistent contraception use (32.3%, *n* = 30), followed by concerns about contraceptive side effects (22.6%, *n* = 21) (Figure 3). In addition, 12.9% of participants (*n* = 12) reported that their partner would not allow them to use contraception or might hurt them if they did. Approximately three-quarters (76.3%, *n* = 142) felt that contraception was easy to obtain, with the majority obtaining it free of charge (67.2%, *n* = 80) at their local clinic (46.5%, *n* = 59). While most participants reported that they were satisfied with the knowledge and skills (78.1% *n* = 100) and attitudes (80.5%, *n* = 103) of their contraception providers, 25.8% reported having unmet contraceptive needs (*n* = 33).

### Emergency contraception

The prevalence of emergency contraception use was 44.1% (*n* = 82), with most having obtained it within 1–2 days after unprotected sexual intercourse (97.6% *n* = 80).

### Contraceptive counselling and prescription

The prevalence of contraceptive counselling reported by participants was 25.3% (*n* = 47), with less than one-third of participants indicating that their MHCP had informed them of the potential teratogenicity of their psychiatric medication (31.2%, *n* = 58) and only 20 (10.8%) participants having been prescribed contraception by them. Evidence of contraceptive counselling and prescription in the participants’ charts was low, with 10.2% documenting counselling (*n* = 19) and 5.9% (*n* = 11) having been prescribed contraception. The majority (59.7%, *n* = 111) reported that they would prefer to collect contraception at their psychiatric facility.

### Clinical characteristics

#### Primary psychiatric diagnosis

Bipolar and related disorders (36.6%, *n* = 68), depressive disorders (32.8%, *n* = 61) and schizophrenia spectrum disorders (24.7%, *n* = 46) were the most common diagnoses. Other diagnoses, including trauma and stressor; anxiety; neurocognitive, somatic symptoms and attention-deficit hyperactivity disorders (ADHD), collectively account for a small proportion of the sample (14.5%, *n* = 27).

Nearly a quarter of participants had a comorbid diagnosis of a personality disorder or traits (24.2%, *n* = 45), while 8.1% were diagnosed with a substance use disorder (*n* = 15). Regarding STIs, 16.1% had a documented positive diagnosis of HIV (*n* = 30), 50% were negative (*n* = 93) and 33.9% (*n* = 63) had an unknown HIV status, while syphilis was positive in one participant (0.5%).

#### Medication

The majority were prescribed an antipsychotic (83.3%, *n* = 155), while 51.1% (*n* = 95) were prescribed antidepressants, with 118 (63.4%) being prescribed any teratogen. Mood stabilisers were prescribed in over half of the sample (54.3%, *n* = 101), 34.4% participants were prescribed a teratogenic mood stabiliser (*n* = 64), 38.2% were prescribed a benzodiazepine (*n* = 71) and 10 participants were prescribed methylphenidate (5.4%).

#### Associations between socio-demographic, reproductive characteristics and consistency of contraception use

Participants attending the outpatient department used contraception more consistently than inpatients (*p* = 0.018) (Table 2), with English language also being associated with more consistent use (*p* = 0.044). No statistically significant association was found between consistency of contraceptive use and level of education (*p* = 0.805), employment status (*p* = 0.521) or unplanned pregnancy (*p* = 0.402). Single participants used contraceptives less consistently (*p* = 0.049), while prior pregnancy was significantly associated with consistent contraceptive use (*p* = 0.046). Women consistently using contraception had significantly less children on average (1.88, SD = 0.95) compared to those with inconsistent use (1.95, SD = 0.91). Women with a history of forced sex used contraceptives more consistently (*p* = 0.046), with no association being found between use consistency and desiring future children (*p* = 0.077).

#### Associations between contraceptive methods, counselling and consistency of contraception use

Hormonal contraceptive (*p* ≤ 0.001) and long-acting method use (*p* ≤ 0.001) were both significantly associated with consistent contraception use (Table 3). Participants who reported having received contraceptive counselling by their MHCP used contraception more consistently than those who had not received counselling (*p* = 0.018). Those who had received information on the teratogenicity of their psychotropics used contraceptives more consistently (*p* = 0.007).

**TABLE 3:** Comparison of contraceptive characteristics of inconsistent and consistent contraceptive users.

Variable	Overall (*N* = 186)		Inconsistent users (*N* = 93)		Consistent users (*N* = 93)		*P*
	*n*	%	*n*	%	*n*	%	
**Contraception**	-	-	-	-	-	-	-
Hormonal	72	59.0	11	36.7	61	66.3	< 0.001*
Non-hormonal	50	41.0	19	63.3	31	33.7	-
Long-acting	35	28.7	2	6.7	33	35.9	< 0.001*
Short-acting	87	71.3	28	93.3	59	64.1	-
**Counselling by MHCP**	-	-	-	-	-	-	-
Contraception	47	25.3	16	17.2	31	33.3	0.018*
Teratogenicity	58	31.2	20	21.5	38	40.9	0.007*
**Contraception prescribed**	20	10.8	6	6.5	14	15.1	0.096

MHCP, mental health care provider.

* , *p*-value statistically significant (*p* ≤ 0.05).

#### Associations between medication, diagnosis and consistency of contraception use

Depressive disorders (*p* = 0.012) and antidepressant use (*p* = 0.04) were both significantly associated with consistent contraception use (Table 4). Participants with schizophrenia spectrum disorders (*p* < 0.001) and antipsychotic prescriptions used contraceptives less consistently (*p* = 0.017). No statistically significant difference was found between overall teratogenic medication prescription and the consistency of contraception use (*p* = 0.447).

**TABLE 4:** Comparison of clinical characteristics of inconsistent and consistent contraceptive users.

Variable	Overall (*N* = 186)		Inconsistent contraception (*N* = 93)		Consistent contraception (*N* = 93)		*P*
	*n*	%	*n*	%	*n*	%	
**Primary diagnosis**	-	-	-	-	-	-	-
Schizophrenia spectrum	46	24.7	34	36.6	12	12.9	< 0.001*
Bipolar and related disorders	68	36.6	31	33.3	37	39.8	0.447
Depressive disorder	61	32.8	22	23.7	39	41.9	0.012*
Other	27	14.5	11	11.8	16	17.2	0.406
**Co-morbidity**	-	-	-	-	-	-	-
Personality Disorder/trait	45	24.2	17	18.3	28	30.1	0.086
Substance use	15	8.1	10	10.8	5	5.4	0.281
Other	35	18.8	17	18.3	18	19.4	1.000
**Psychotropics**	-	-	-	-	-	-	-
Antipsychotic	155	83.3	84	90.3	71	76.3	0.017*
Antidepressant	95	51.1	40	43.0	55	59.1	0.040*
Lamotrigine	55	29.6	26	28.0	29	31.2	0.748
**Teratogenic mood stabiliser**	61	32.8	35	37.6	26	28.0	0.211
Na Valproate	43	23.1	26	28.0	17	18.3	0.164
Carbamazepine	5	2.7	3	3.2	2	2.2	1.000
Lithium	8	4.3	5	5.4	3	3.2	0.721
Topiramate	8	4.3	4	4.3	4	4.3	1.000
**Benzodiazepine**	71	38.2	37	39.8	34	36.6	0.763
**Methylphenidate**	10	5.4	5	5.4	5	5.4	1.000
**Any Teratogen**	118	63.4	62	66.7	56	60.2	0.447

* , *P*-value = statistically significant (*p* ≤ 0.05).

Within the group of participants who were prescribed a teratogenic mood stabiliser (*n* = 61), a higher percentage reported inconsistent contraceptive use, although this finding was not statistically significant (*p* = 0.211). Participants prescribed teratogens were significantly more likely to have documented evidence of contraceptive counselling than those not prescribed a teratogen (*p* = 0.002).

## Discussion

### Prevalence of contraception use

This study found that the prevalence of current contraceptive use among women with MI was 59.1%, which is consistent with local and global averages,^14,15,26^ with only 50% reporting consistent use. The perfect use of contraceptives is essential for effective family planning, as erratic use increases the risk of unplanned pregnancies and exposure to teratogenic medication.^16,34^ Factors associated with the consistency of contraceptive use were explored to identify areas for possible intervention.

### Socio-demographic, diagnostic and reproductive factors

Socio-demographic factors associated with increased consistency of contraception use were outpatient status and proficiency in English, suggesting that psychiatric outpatients may possess decision-making capacities that inpatients lack because of the severity of their MI.^35^ Participants with depressive disorders, commonly managed in outpatient settings, had more consistent contraceptive use compared to those treated for severe relapses of schizophrenia and bipolar disorders. Research highlights MHCP’s reluctance to initiate or maintain contraceptives during inpatient psychiatric stays, often deeming it non-essential and deferring to after discharge.^24^ However, contraceptive prescription during admissions can establish a contraceptive routine and address patient concerns timeously.^24^

The reasons behind the higher consistency of contraception use among English-speaking participants remain unclear, as this correlation was not observed within racial categories or linked to education. One possible explanation may be related to patient–provider interactions that can influence contraceptive uptake, including language and culture.^36^ Minority groups lacking proficiency in English often experience shorter consultations, receive less information and have difficulty understanding English-speaking health care providers. Disparities in the quality of health care and communication received by non-English-speaking patients could diminish their contraceptive use.^2,36^ Although previous studies have linked education with contraceptive use, this association was not found in our study,^15,17,37^ which may be related to the fairly high levels of education among participants and the small, homogenous sample.

Our findings also indicated that single participants used contraceptives less frequently, which aligns with other local research,^26^ though the impact of relationship status on contraceptive use varies in the literature.^38^ Some studies report that women in casual relationships use consistent contraception less than those in long-term relationships, while others suggest that contraception is inversely proportional to the degree of suspected commitment in the relationship.^38,39^ As the majority of our study population was single, the risk of unplanned pregnancies and potential teratogenicity is high. Women with a prior pregnancy were also observed to use contraceptives more consistently. This was proved through a Ghanaian cross-sectional study finding that participants with a history of prior pregnancy were over two times more likely to use contraceptives than those without.^40^ However, unplanned pregnancy was not significantly associated with contraceptive use in this study.

### Contraceptive methods

Short-acting methods of contraception, particularly male condoms and hormonal injectables, were used by the majority of participants, despite long-acting methods being used more consistently (*p* ≤ 0.001). This aligns with both local and international data, indicating that women in lower-income countries rely on short-acting methods with higher failure rates than women in the first world.^15,16^ While condoms are effective at preventing pregnancy and reducing the risk of STIs when used properly, user error, inconsistent use and, especially in the context of MI, partner willingness to use can impact on the method’s success.^18^ Women with MI should ideally use long-acting contraceptive methods to minimise non-adherence and reduce the likelihood of unplanned pregnancies.^13^

Hormonal methods have received negative publicity in the psychiatric context because of their potential link with mood disorders, although this has largely been disproven.^21,22^ In this study, women with MI were more likely to use hormonal methods consistently than non-hormonal methods (*p* ≤ 0.001). This suggests that discontinuation because of suspected hormone-induced side effects is not significant, despite some literature suggesting otherwise.^13,22^ Women in this sample used injectable contraceptives as often as male condoms, possibly because of their availability and affordability. The uptake of long-acting methods could improve if they are freely stocked and promoted by MHCPs. Of concern is that only 17 participants used dual methods of contraception, and women with MI are at high risk of contracting STIs, highlighting the need for counselling to enhance preventative measures.^5,6,26^

### Barriers to contraception

Aside from sexual inactivity, concerns around contraceptive side effects were identified as a significant barrier to their use, and the literature indicates that misconceptions around contraceptive side effects greatly affect their uptake.^20^ Comprehensive contraceptive counselling is, therefore, essential to address these concerns and guide patients to select the most suitable method while minimising the risk of polypharmacy and its association with non-adherence, given their use of psychotropic medications.^13,41^ The third most common barrier was fear of a partner, again reflecting the vulnerability of women with MI to reproductive coercion.^10^ Longer-acting methods, such as the IUCD, could provide discreet contraception that remains undetectable by partners and should be discussed in the counselling process.

### Contraceptive counselling

Over a quarter of participants felt that their contraceptive concerns were not adequately addressed by their current health care provider and used contraception more consistently if they felt their concerns were being addressed (*p* = 0.003). The prevalence of contraceptive counselling and prescription in the study were both very low, which is consistent with the findings of Galvin et al.,^26^ where MHCP’s inadequate counselling directly impacts on the uptake. Both contraceptive (*p* = 0.018) and teratogenicity counselling (*p* = 0.007) were significantly associated with contraceptive use, demonstrating that time invested in counselling can directly improve contraceptive usage in women with MI, reducing the impact of unplanned pregnancy and exposure to teratogens.^7,32^

The notably low prescription of contraception can partly be attributed to the fact that this psychiatric facility only provides psychiatric medication to outpatients, while non-psychiatric medications need to be obtained at another facility. Although this practice is common at many psychiatric hospitals, it may highlight a service gap, as nearly 60% of participants expressed a preference for obtaining their contraception at their psychiatric facility, which is supported in the literature.^18^

Over one-third of participants were prescribed a teratogenic mood stabiliser, and nearly two-thirds were prescribed any teratogen. Despite these high rates, no statistically significant association was observed between contraceptive use and teratogenic medication prescription. Women with MI require individualised psychiatric care, balancing the teratogenic risks of psychotropics with the benefits of treating psychiatric symptomatology.^27^ While teratogenic medications are occasionally required for the patient’s well-being, efforts to ensure adequate contraception with lower failure rates can help mitigate the risks of these drugs, necessitating active contraceptive counselling and prescription.^27^

### Reproductive factors

Approximately two-thirds of all pregnancies reported by participants were unplanned (65.9%, *n* = 81), which exceeds global and local prevalence rates.^8^ A large, multi-centre cross-sectional study examining unintended pregnancy in nearly 35 000 pregnant, South African women found a prevalence of 51.6%.^42^ This is lower than the 65.9% observed in our study, corroborating that women with MI are at an increased risk of unintended pregnancy.

The literature describes the inherent vulnerability of women with MI to sexual coercion, rape and the transmission of HIV and STIs.^5,6,10^ The lifetime prevalence of sexual violence in South Africa is estimated at 24.9%.^43^ A South African study on the epidemiology of rape found that one in four women in Rustenberg, Gauteng Province, was a victim of sexual violence.^43^ In our study, the prevalence of forced sex was 35.5% (*n* = 66), equating to more than one in three participants having been subjected to sexual violence. This high prevalence underscores that women with MI remain at significant risk despite advocacy efforts and campaigns against gender-based violence. Proactive contraceptive counselling and prescription can help to mitigate the numerous vulnerability factors associated with reproductive-age women with MI.^10,13^

### Strengths and limitations

This study identified a positive association between contraceptive counselling by MHCPs and the consistency of contraceptive use in women with MI, which contributes to the limited knowledge base on women’s mental and reproductive health in South Africa, guiding further research and interventions to address their unmet reproductive needs.

Limitations of the study include the lack of a standardised, validated survey tool, which limits the reliability, generalisation and reproducibility of the data, as well as the possible introduction of selection bias using convenience sampling. Participants were predominantly English speaking with a grade 12 or tertiary education level, which is unlikely to reflect the demographic characteristics of the local population. As the survey was completed by self-reporting of participants, recall and information bias may have been introduced. Missing and unknown data from participant charts limited the analysis of certain categories of data. Owing to the cross-sectional nature of the study, no causal inferences were possible, while a larger sample size would have improved the power of the statistical findings.

## Recommendations

This study highlights the insufficient contraceptive use and the lack of contraceptive counselling among women with MI. Further research is needed in diverse populations and community samples with larger sample sizes, focusing on both qualitative insights and a deeper exploration of the barriers to contraceptive use. It is crucial to educate MHCPs on reproductive health matters and to foster collaboration between psychiatric, obstetric and gynaecological services to improve the care provided by both sectors. Public health campaigns promoting contraceptive use, along with the integration of reproductive and mental health services, could help to address the unmet contraceptive needs of this vulnerable population.

## Conclusion

There is inconsistent contraceptive use and inadequate contraceptive counselling in women with MI, with high rates of unplanned pregnancies, forced sex and teratogen prescription, which, in the absence of consistent contraception, reflect the deficits in care in this at-risk group. Mental health care providers have a responsibility to counsel patients on the importance of contraception, especially in the context of teratogen prescription. This study provides evidence that contraceptive counselling can improve the consistency of contraception use by women with MI, ultimately reducing the incidence of unplanned pregnancies and their long-term sequalae. Integrated women’s reproductive and mental health services could address the unmet contraceptive needs of this vulnerable group. Future studies should explore the efficacy of MHCP-driven contraceptive counselling to improve contraceptive uptake in women with MI and determine ways to address their barriers to contraceptive use.
